# Influence of Body Position Changes on Diaphragmatic Excursion Assessed by Ultrasonography in a Healthy Population

**DOI:** 10.3390/jfmk11010064

**Published:** 2026-01-31

**Authors:** Leonardo Arzayus-Patiño, Jorge Enrique Daza-Arana, Santiago Vásquez Cartagena, Carolina Villamizar, Juan Meléndez Diaz, Diego Fernando Muñoz-Escudero

**Affiliations:** 1 Programa de Fisioterapia, Facultad de Salud, Universidad Santiago de Cali, Cali 76000, Colombia; 2 Doctorado en Ciencias Aplicadas, Facultad de Ciencias Básicas, Universidad Santiago de Cali, Cali 76000, Colombia; 3 Centro de Ciencias Básicas, I.U. Escuela Nacional del Deporte, Universidad del Rosario, Bogota 110110, Colombia

**Keywords:** diaphragmatic excursion, ultrasonography, body position, respiratory function, diaphragm assessment

## Abstract

**Background:** The diaphragm is the primary respiratory muscle, and its proper function is essential for efficient breathing. Respiratory muscle weakness is a common complication that can hinder the withdrawal of mechanical ventilation. This weakness not only negatively affects patients’ quality of life but also represents an economic challenge for healthcare systems, as it significantly increases medical costs due to prolonged hospitalization and the need for additional procedures to manage associated complications. Ultrasonography has emerged as a precise technique for assessing diaphragmatic function through measurements such as diaphragmatic excursion and thickening fraction, with the right hemidiaphragm being the most suitable for evaluation. However, several studies have shown that diaphragmatic ultrasound measurements vary considerably in both healthy individuals and patients, mainly due to the lack of standardization of body position during assessment. Therefore, it is necessary to investigate how patient posture influences diaphragmatic ultrasound measurements in order to standardize protocols, improve diagnostic accuracy, and support reliable clinical decision-making. We employed ultrasonography to determine the influence of changes in body position on diaphragmatic excursion in a healthy population from the city of Cali. **Methods:** A descriptive cross-sectional study was conducted in 36 healthy adults aged 18 to 65 years, distributed into sex and age groups. Diaphragmatic excursion was assessed using a 3.5–5 MHz ultrasound transducer. Participants were evaluated in five body positions: supine at 0°, and head-of-bed inclinations of 30°, 45°, 70°, and 90°. **Results:** A progressive increase in diaphragmatic excursion was observed from the supine position (0°) up to 70° inclination. The 70° inclination showed the greatest diaphragmatic mobility as measured by ultrasonography. This finding suggests the existence of an optimal intermediate position in which biomechanical conditions and intra-abdominal pressure allow more efficient diaphragmatic contraction. **Conclusions**: The results of this study demonstrate that changes in body position significantly influence diaphragmatic excursion in healthy individuals, with a trunk inclination of 70° yielding the greatest diaphragmatic mobility. These findings support the importance of considering body posture as a key determinant in the functional assessment of the diaphragm using ultrasonography.

## 1. Introduction

The diaphragm is the primary respiratory muscle, and its proper function is essential for efficient breathing. In hospitalized patients, particularly those in intensive care units, respiratory muscle weakness is a common complication that can hinder the withdrawal of mechanical ventilation [1]. This weakness not only negatively affects patients’ quality of life but also represents an economic burden for healthcare systems, as it significantly increases medical costs due to prolonged hospitalization and the need for additional medical procedures to manage associated complications [2].

To mitigate the effects of muscle weakness, appropriate assessment of both respiratory and peripheral musculature is essential. Respiratory muscle strength is commonly evaluated using measurements such as Maximum Inspiratory Pressure (MIP) and Maximum Expiratory Pressure (MEP), while peripheral muscle strength can be assessed using tools such as handgrip strength testing and the Medical Research Council muscle strength scale (MRC-SS) [3]. These measurements provide objective information on functional muscle status and allow healthcare professionals to design personalized rehabilitation programs and make informed clinical decisions aimed at reducing muscle weakness. The implementation of these evaluations in clinical practice is fundamental for optimizing recovery and reducing length hospital of stay.

With technological advances and the need for more precise assessments, the evaluation of respiratory musculature has become increasingly specific. Although tools such as MIP and MEP are useful, they do not isolate or directly assess the diaphragm, which is the main muscle involved in respiration. In this context, ultrasonography has emerged as a valuable technique, enabling direct assessment of diaphragmatic excursion and the diaphragmatic thickening fraction [4]. These ultrasound-derived measurements provide a more accurate evaluation of diaphragmatic function and allow a more precise estimation of muscle recruitment and force generation, making them relevant indicators of diaphragmatic performance, particularly in critically ill patients [5].

The use of ultrasonography to assess the diaphragm has become an important resource in clinical decision-making, especially for the identification of impairments in diaphragmatic muscle performance. However, several studies have demonstrated that diaphragmatic ultrasound measurements show considerable variability in both healthy populations [6] and clinical populations [7]. This variability suggests that methodological differences in diaphragmatic ultrasonography may substantially influence measurements and compromise their clinical interpretation.

Parada et al. [8] conducted a systematic review on diaphragmatic ultrasonography as a predictor of weaning and highlighted substantial heterogeneity in the protocols of the included studies. Specifically, the authors reported variations in body positioning or, in some cases, the absence of clear specification of the posture used, identifying this factor as an important contributor to variability in diaphragmatic measurements. Similar limitations were identified in the EXODUS consensus [9], which failed to reach agreement on standardized patient and transducer positioning, despite evidence confirming the influence of posture on diaphragmatic ultrasound measurements. Therefore, standardization of assessment protocols is necessary to ensure measurement reliability and comparability, which are fundamental for clinical decision-making.

Several studies have demonstrated that body position significantly influences diaphragmatic ultrasound measurements, particularly when comparing supine, seated, and standing postures. Hellyer et al. [10] reported clinically relevant variations in diaphragmatic thickness and excursion across different postures, highlighting the role of gravity and postural changes in diaphragmatic morphology and function. Similarly, Brown et al. [11]. Showed that body position modifies ultrasonographic measurements of diaphragmatic contractility, emphasizing the mechanical impact of posture on diaphragmatic behavior. Nevertheless, within the range of positions commonly feasible in hospitalized patients, from the supine position to sitting at 90°, it remains unclear which posture provides the most reliable measurements of diaphragmatic excursion. In this context, it is relevant to investigate how changes in body position influence diaphragmatic ultrasound measurements, particularly diaphragmatic excursion. This knowledge is essential not only to improve measurement accuracy and advance protocol standardization but also to ensure that healthcare professionals can rely on these measurements for clinical decision-making. Based on this need for standardization, the following research question is proposed: What is the influence of changes in body position on diaphragmatic excursion assessed by ultrasonography in a healthy adult population from the city of Cali?

## 2. Materials and Methods

### 2.1. Study Design and Participants

A descriptive cross-sectional study was conducted, with data collected at a single time point to describe and analyze the influence of body position on diaphragmatic excursion in a healthy population from the city of Cali. Data collection was carried out between January and August 2025.

The study population was recruited by convenience sampling and consisted of healthy adults residing in Cali, aged between 18 and 65 years, distributed into sex and age groups. The sample size calculation was based on a statistical power of 80% and a significance level of 5%. It was estimated that approximately 16 participants per sex group would be required to detect a minimum difference of 0.1 cm in diaphragmatic excursion. This minimum difference was defined based on previous studies with similar characteristics [10], assuming a standard deviation of 0.1 cm. The sample size was calculated using the following formula for the comparison of two means:

$$n\;=\;\backslash frac\left\{{\left(Z_{\left\{\frac{\alpha }{2}\right\}}+\;Z_{\left\{\beta \right\}}\right)}^{2}\cdot 2\;\cdot \sigma^{2}\right\}\left\{\Delta^{2}\right\}$$

### 2.2. Sample Size and Participants

The sample size was estimated using the formula for comparison of means, considering a two-sided confidence level of 95% (Zα/2 = 1.96), a statistical power of 80% (Zβ = 0.84), an expected standard deviation (σ) of 0.1 cm, and a minimum detectable difference (Δ) of 0.1 cm. The assumed standard deviation was based on previously reported ultrasound data and pilot observations available during the planning phase of the study [10], acknowledging the physiological variability inherent to diaphragmatic excursion. Based on this calculation, a total of 32 participants was required.

Given the exploratory nature of the study and to allow stratification by sex and age, a final sample of 36 healthy participants was included, evenly distributed as 18 men and 18 women.

The study sample consisted of apparently healthy voluntary participants selected from the city of Cali, Colombia. Participant selection was based on the following inclusion criteria: adults older than 18 years of both sexes, apparently healthy, without a diagnosis of significant chronic diseases that could affect diaphragmatic function, and without mental, cognitive, visual, or auditory impairments that could compromise understanding of instructions or proper execution of the ultrasound maneuvers.

Exclusion criteria included pregnant women; individuals with a history of dyspnea of unknown origin; individuals with neuromuscular diseases or peripheral neuropathy; and individuals with skin sensitivity, irritation, or conditions that could interfere with the safe and adequate application of the ultrasound gel required for the examination.

Given that the right hemidiaphragm provides greater accessibility and improved image definition during ultrasonographic assessment due to the hepatic acoustic window, only this side was evaluated [2,12]. The liver offers a homogeneous and stable acoustic window that facilitates consistent visualization of diaphragmatic motion in both B-mode and M-mode, whereas assessment of the left hemidiaphragm is often limited by gastric air and the spleen, which provides a less favorable acoustic window. This approach was adopted to enhance measurement reproducibility and ensure methodological standardization across participants.

### 2.3. Ultrasound Measurements and Reliability

Ultrasound measurements were performed independently by two physiotherapists specialized in cardiopulmonary physiotherapy. Both evaluators had certified training and prior experience in diaphragmatic ultrasonography, ensuring measurement accuracy and objectivity. To minimize observation bias, evaluators were blinded to each other’s measurements and to their own previous records throughout the entire process.

To assess measurement consistency, intraobserver and interobserver reliability analyses were conducted prior to the start of the study using the intraclass correlation coefficient (ICC), applying a two-way mixed-effects model with absolute agreement. Reliability testing was performed in an independent group of 20 healthy subjects who were not included in the final study sample. Both evaluators performed repeated measurements of diaphragmatic excursion following the same standardized protocol.

Intraobserver reliability was assessed by comparing repeated measurements obtained by the same evaluator in the same subjects, whereas interobserver reliability was determined by comparing measurements independently obtained by both evaluators. Measurement consistency was analyzed using ICC, showing excellent reliability levels. For diaphragmatic excursion, the intraobserver ICC was 0.97 (95% CI: 0.94–0.99), and the interobserver ICC was 0.95 (95% CI: 0.91–0.98), ensuring reproducibility and validity of the measurements.

Based on the reliability analysis, the standard error of measurement (SEM) and the minimal detectable change at the 95% confidence level (MDC95) were calculated to further support the interpretation of diaphragmatic excursion variability. Using a standard deviation of 0.28 cm derived from the reliability sample, the SEM was 0.05 cm for intraobserver measurements and 0.06 cm for interobserver measurements. The corresponding MDC95 values were 0.14 cm and 0.17 cm, respectively. These parameters indicate the minimum change required to exceed measurement error and provide additional methodological and clinical context for the ultrasonographic assessment of diaphragmatic excursion.

A pilot study was conducted in 10 healthy individuals to determine the approximate time required to complete the measurements, verify proper equipment functioning, assess the adequacy of the data collection forms, and ensure participant understanding of the instructions. Following the pilot study, necessary adjustments were made to finalize the standardized operating procedures. Individuals participating in the pilot study were not included in the final sample.

### 2.4. Recruitment and Preparation

Participants were recruited through social media platforms and email invitations that included detailed study information and the approval letter from the Ethics Committee of Universidad Santiago de Cali. Individuals meeting the selection criteria were scheduled at the simulated hospital clinic, where informed consent was read and signed prior to data collection.

Before evaluation, participants were instructed to follow specific recommendations to optimize measurement validity: maintain fasting for at least three hours before assessment; avoid vigorous physical activity (>5 METs) during the 12 h prior to evaluation; refrain from smoking during the 12 h before assessment; and wear comfortable clothing allowing adequate exposure of the evaluation area.

Once these conditions were verified, sociodemographic data and baseline measurements of weight and height were recorded, followed by diaphragmatic ultrasound assessment. Measurements were conducted during morning or afternoon sessions according to participant availability and the assigned location.

### 2.5. Ultrasound Protocol

The ultrasound examination was performed in a quiet, well-ventilated environment with comfortable ambient temperature. To ensure image quality, participants were asked to wear clothing that allowed abdominal exposure (men without shirts and women wearing a sports top) and to remove any metallic objects that could interfere with the transducer. To minimize external influences on the respiratory pattern, only the evaluator and the participant were present during image acquisition.

To evaluate the influence of posture, participants progressively adopted five head-of-bed inclination positions: supine at 0°, followed by 30°, 45°, 70°, and seated at 90°. Each angle was accurately verified and recorded using an angle measurement device and goniometry to ensure positional standardization. After each positional change, a two-minute waiting period was allowed to facilitate physiological accommodation and stabilization of ventilation and intra-abdominal pressure. During this period, respiratory frequency was continuously monitored using a multiparameter monitor to confirm stability before measurements were initiated.

The measurement area was located in the right subcostal region. A convex transducer (2–5 MHz) was placed between the midclavicular line and the anterior axillary line, using the liver as an acoustic window. Image depth was individually adjusted in each participant to ensure optimal visualization of the right hemidiaphragm, taking into account interindividual differences in the distance between the skin, subcutaneous adipose tissue, and the hepatic acoustic window. The hyperechoic diaphragmatic line was identified in B-mode (2D) to visualize diaphragmatic motion. Participants were instructed to breathe calmly without additional effort or speaking during the procedure. No respiratory training or voluntary control of breathing was applied, in order to preserve resting physiological conditions. All measurements were performed during quiet tidal breathing, and image acquisition was initiated only when a stable respiratory pattern was observed. System gain was adjusted to achieve optimal contrast between the diaphragm and surrounding structures and was kept constant throughout the measurements.

**Figure 1:**
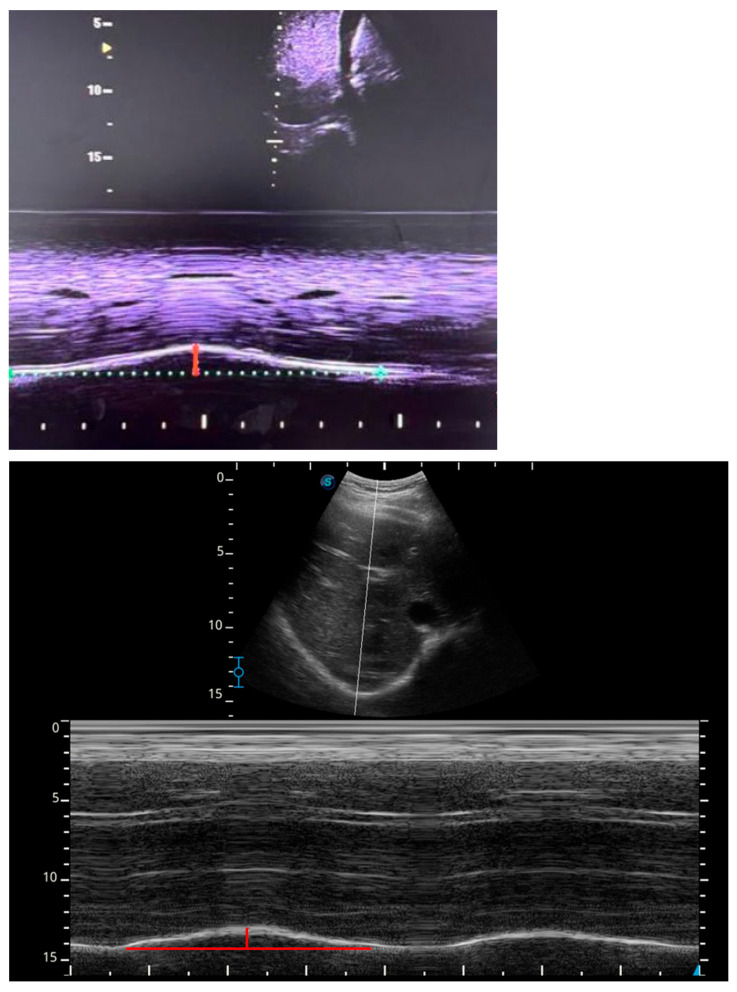
Representative ultrasound image showing diaphragmatic excursion assessed using M-mode. The grayscale B-mode image (upper panel) illustrates probe positioning and diaphragm visualization, while the M-mode tracing (lower panel) depicts diaphragmatic motion over time. The colored horizontal line represents the baseline position of the diaphragm at end-expiration, and the colored vertical line indicates the maximal diaphragmatic displacement during inspiration. Diaphragmatic excursion was calculated as the distance between baseline and maximal inspiratory displacement.

The ultrasound mode was then switched to M-mode to visualize diaphragmatic movement. The M-mode sweep speed was maintained using the standardized default setting of the ultrasound system, in accordance with international expert consensus recommendations. Once a clear image was obtained, the image was frozen, and diaphragmatic excursion was measured in centimeters from the baseline to the point of maximal displacement. In each body position, three consecutive measurements were obtained and subsequently averaged for statistical analysis. A two-minute stabilization period was observed after each positional change to allow physiological accommodation and stabilization of ventilation and intra-abdominal pressure, after which respiratory frequency stability was reconfirmed before initiating the subsequent measurement (Figure 1).

To ensure the quality of the recorded information, a computer dedicated exclusively to this purpose was used. The principal investigator performed random checks to verify consistency between the original ultrasound data, the corresponding data collection forms, and the database. Any detected errors were corrected immediately.

In addition, a second investigator, independent from the principal investigator and not involved in the measurement process, conducted random reviews and quality control checks of the data collection forms to ensure data accuracy prior to database entry. Subsequently, the entered data were cross-checked against the original records to confirm consistency.

After completion of the quality control process, the data was entered into Microsoft Excel 2020 and then exported to SPSS for analysis. Statistical analyses were performed using SPSS version 29.0 (IBM Corp., Armonk, NY, USA).

This study was classified as minimal-risk research in accordance with Resolution 008,430 of 1993 issued by the Colombian Ministry of Health (currently the Ministry of Social Protection). In addition, strict adherence to international ethical principles for research involving human subjects, as outlined in the Declaration of Helsinki, was ensured. The study was approved by the Ethics Committee of Universidad Santiago de Cali (approval code 20241115; approval date: 15 November 2024).

### 2.6. Data Analysis

A descriptive analysis was first conducted to characterize the study population and to explore the distribution of all variables. Continuous variables were summarized using measures of central tendency (mean or median) and dispersion (standard deviation, minimum and maximum values, and interquartile range) as appropriate. Categorical variables were described using absolute frequencies and percentages. The normality of quantitative variables was assessed using the Shapiro–Wilk test.

Subsequently, to appropriately account for the repeated-measures design and to evaluate the influence of body position on diaphragmatic excursion, a linear mixed-effects model was applied. Body position (0°, 30°, 45°, 70°, and 90°) was included as a within-subject factor, and sex (male, female) as a between-subject factor, with participant included as a random effect. This approach allowed simultaneous evaluation of the main effects of body position and sex, as well as their interaction (sex × position).

When significant effects were identified, post hoc pairwise comparisons were performed using Bonferroni adjustment. Statistical significance was set at *p* < 0.05. All statistical analyses were performed using SPSS version 29.0 (IBM Corp., Armonk, NY, USA).

## 3. Results

Between May and July 2025, measurements were performed in a sample of 36 healthy individuals, of whom 50% were women. The mean age of the participants was 41.2 ± 14.2 years, and the mean body mass index (BMI) was 26.6 ± 3.9 kg/m^2^. According to the International Physical Activity Questionnaire (IPAQ), all participants presented low to moderate levels of physical activity. Chest circumference showed statistically significant differences, with higher mean values observed in males (Table 1).

**Figure 2:**
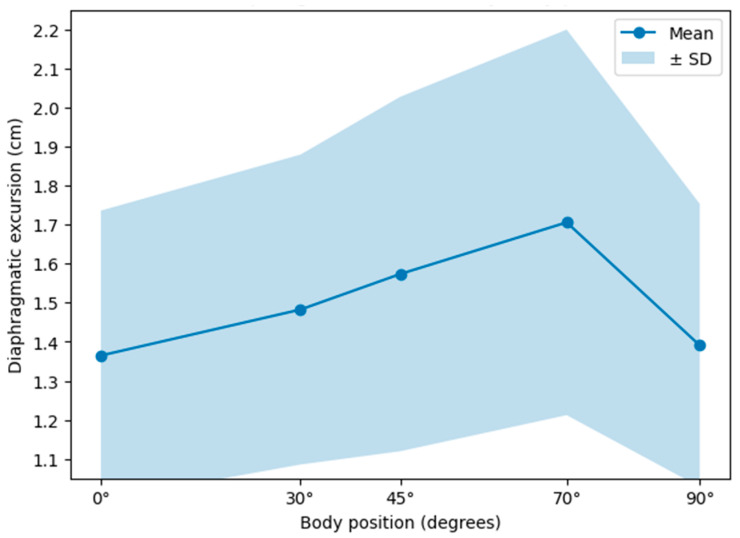
Mean diaphragmatic excursion and standard deviation (SD).

Diaphragmatic excursion showed a pattern of progressive increase with changes in body position, reaching its highest value at the 70° position. From the supine position (0°), a sustained increase was observed through the intermediate positions (30° and 45°), followed by a marked decrease at the 90° position (see Table 2 and Figure 2).

### 3.1. Sex-Based Analysis of Diaphragmatic Excursion

When diaphragmatic excursion was analyzed according to sex, distinct patterns of behavior were observed.

**Figure 3:**
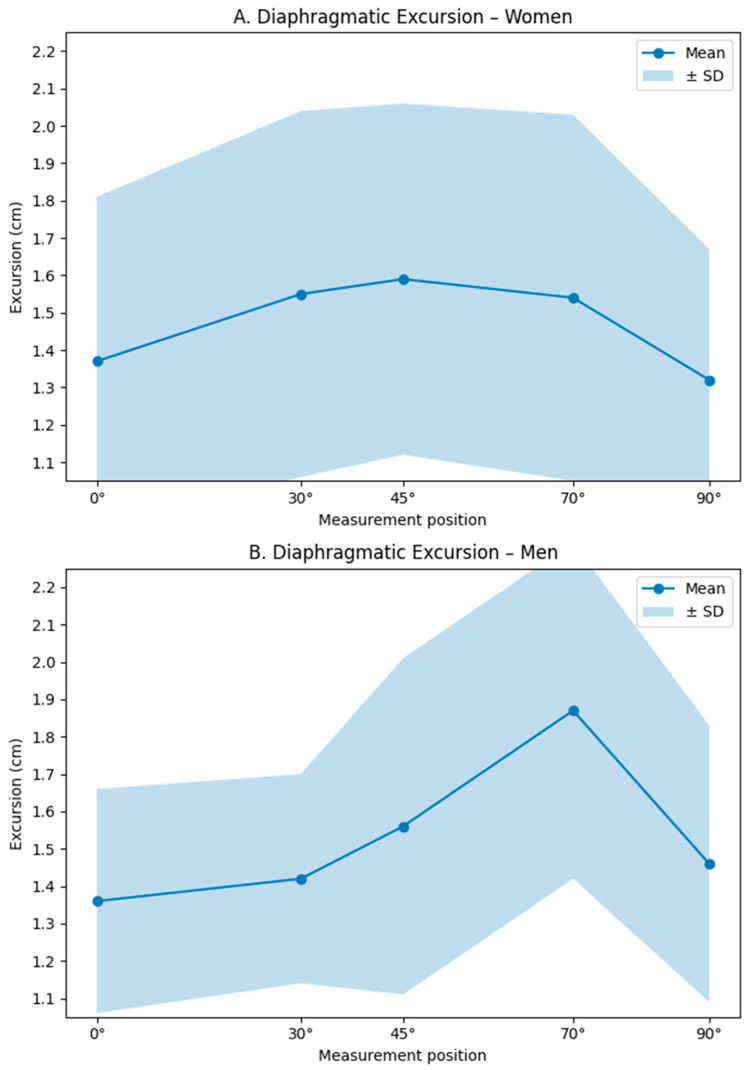
(**A**) Differences in diaphragmatic excursion across body positions in women. (**B**) Differences in diaphragmatic excursion across body positions in men.

In women, diaphragmatic excursion exhibited a non-linear pattern, with the highest values observed at 45°, followed by 30° and 70° (Table 3; Figure 3A).

In men, diaphragmatic excursion demonstrated a more linear and progressive pattern as trunk inclination increased. The greatest diaphragmatic excursion was observed at 70°, followed by 45° and 90° (Table 3 and Figure 3B).

### 3.2. Results—Mixed-Effects Model Analysis

A linear mixed-effects model was applied to evaluate the influence of body position and sex on diaphragmatic excursion, with participant included as a random effect to account for repeated measurements.

The model showed a significant main effect of body position on diaphragmatic excursion. Compared with the supine position (0°), trunk inclination at 45° was associated with a statistically significant increase in diaphragmatic excursion (β = 0.216 cm; *p* = 0.017), representing the position with the greatest overall effect. The 30° and 70° positions showed non-significant increases relative to the supine position, while no significant differences were observed at 90°.

No significant main effect of sex on diaphragmatic excursion was identified (*p* = 0.920). However, a significant interaction between sex and body position was observed at 70°, indicating that men exhibited significantly greater diaphragmatic excursion than women at this trunk inclination (β = 0.348 cm; *p* = 0.007).

## 4. Discussion

The present study aimed to determine the influence of changes in body position on diaphragmatic excursion assessed by ultrasonography in a healthy population from the city of Cali. The results demonstrated that diaphragmatic displacement varies significantly according to the adopted posture. Based on the mixed-effects model, the 45° trunk inclination showed the greatest overall effect on diaphragmatic excursion, while a sex-dependent effect was observed at 70°, with greater excursion in men. These findings indicate that biomechanically favorable conditions for diaphragmatic displacement depend both on body position and, in specific postures, on sex-related factors.

This finding can be explained from a physiological perspective. The increase in diaphragmatic excursion observed in more upright positions may be related to a reduction in the pressure exerted by the abdominal viscera on the diaphragmatic dome, allowing greater muscle displacement during inspiration. As posture changes from the supine to the seated position, the abdominal contents tend to descend, thereby reducing the opposing load on the diaphragm.

Additionally, the anisotropy theory described by Chen and Boriek in 2023 [13] proposes that the diaphragm is a structurally non-uniform muscle whose mechanical behavior varies according to fiber orientation, load direction, and gravitational influence. This model supports the hypothesis that different degrees of body inclination induce differential patterns of muscle recruitment and mechanical response, which may explain why intermediate trunk inclinations favor diaphragmatic displacement more consistently than extreme positions. Similarly, Hellyer et al. in 2017 [10] demonstrated that diaphragmatic excursion and thickness measured by ultrasonography were greater in seated and standing positions than in the supine position, attributing these findings to gravitational effects and changes in muscle fiber length; however, that study did not include intermediate positions, which limits direct comparison with the present findings.

In this context, the 45° trunk inclination, identified in this study as the position with the greatest overall effect on diaphragmatic excursion, may represent an optimal balance between abdominal pressure, functional diaphragm length, and thoracic axis orientation relative to gravity. By contrast, the 70° position appeared to exert a posture-specific effect modulated by sex, suggesting that biomechanical advantages at higher inclinations may not be uniform across individuals. Consistently, Wang et al. [14]. Reported a strong correlation between abdominal displacement and diaphragmatic excursion in the supine position, demonstrating that diaphragmatic behavior depends not only on the muscle itself but also on its interaction with the thoracic wall and the thoracoabdominal musculoskeletal system. These findings complement existing evidence by highlighting that postural variations directly influence respiratory mechanics and diaphragmatic functional efficiency.

Sex-stratified analysis showed a comparable pattern of diaphragmatic excursion between men and women across most of the body positions evaluated, with the exception of the 70° inclination, where a significant difference was observed. This finding suggests that the effect of posture on diaphragmatic mobility may be modulated by sex-specific morphofunctional characteristics. Previous studies have reported that men tend to exhibit greater diaphragmatic mobility during breathing compared with women [15], which may be related to differences in muscle architecture and thoracoabdominal configuration. In addition, a higher proportion of visceral fat, more frequently observed in men, may influence intra-abdominal pressure and modify the mechanical interaction between the diaphragm and the abdominal viscera [16]. Furthermore, more upright trunk positions facilitate the caudal displacement of abdominal contents, thereby reducing the mechanical load opposing diaphragmatic contraction [11,12]. In this context, an intermediate inclination such as 70° may represent a scenario in which these structural and functional differences become more evident, whereas in lower or more extreme positions (0°, 30°, and 90°), shared mechanical constraints may attenuate sex-related differences. This interpretation is consistent with previous reports that have not identified consistent sex-related differences in diaphragmatic excursion under other postural configurations [10,17]. Therefore, it is suggested that ultrasonographic assessment of diaphragmatic excursion incorporate intermediate positions, such as 45° or 70°, without assuming systematic sex-based differences and avoiding conclusions based on a single posture.

In the remaining positions evaluated, diaphragmatic excursion behaved similarly in men and women, suggesting that under conditions of tidal breathing and in apparently healthy individuals, diaphragmatic excursion does not differ when ventilatory demand is low [7,18]. During tidal ventilation, diaphragmatic excursion is primarily determined by automatic diaphragmatic activation and the passive mechanics of the thoracoabdominal system, factors that tend to be comparable between men and women in the absence of additional ventilatory load [19]. Although anatomical differences between sexes have been described, such as greater lung volumes and increased diaphragmatic muscle thickness in men, these characteristics do not necessarily translate into greater diaphragmatic excursion across all postures, particularly when measurements are performed at rest [2,20]. Likewise, previous studies have reported similar diaphragmatic excursion values between sexes across different body positions, supporting the interpretation that the pattern observed in the present study reflects an expected physiological behavior rather than a lack of methodological sensitivity [10,11].

Differences in socioeconomic status were observed between men and women in the baseline characteristics. Although socioeconomic status has been associated with general health determinants and variables indirectly related to respiratory function, it is unlikely that this factor substantially influenced the main findings of the present study. The primary objective was to evaluate the effect of body position on diaphragmatic excursion using a repeated-measures design, in which each participant served as their own control, thereby minimizing the impact of interindividual factors such as socioeconomic status. All measurements were performed under standardized resting respiratory conditions in apparently healthy participants, supporting the interpretation that the observed postural patterns of diaphragmatic excursion were not driven by socioeconomic differences.

The study by Yamada et al. in 2024 [17], conducted in a Japanese population in the seated position, reported mean diaphragmatic excursion values lower than those observed in the present study (1.39 cm during quiet breathing and 4.4 cm during deep inspiration). These differences may be attributed to population-specific factors such as body mass index (BMI) and anthropometric characteristics. To date, no reference values have been established for the population evaluated in this study, underscoring the importance of generating population- and position-specific standards. This need is fundamental for accurate and contextualized interpretation of results across different groups.

Diaphragmatic excursion in this study was measured using M-mode ultrasonography, which is considered the most effective technique for visualizing diaphragmatic displacement in real time. This methodological choice is supported by international recommendations for diaphragmatic function assessment [4], which establish M-mode as the reference technique for excursion measurement. Furthermore, diaphragmatic excursion measured by ultrasonography has been shown to be particularly sensitive to body position, exhibiting relevant variations depending on patient posture [4]. Although the supine position facilitates subcostal image acquisition, it is associated with greater opposition to diaphragmatic displacement due to abdominal pressure, potentially limiting the observed excursion range.

Likewise, this study reinforces the utility of ultrasonography as a non-invasive diagnostic tool for evaluating diaphragmatic mobility. According to expert consensus on diaphragmatic ultrasonography, this technique demonstrates high sensitivity and specificity and is recommended for use in both critically ill patients and ambulatory populations. Boussuges et al. in 2020 [12] reported that ultrasonography is a valid and sensitive method for assessing diaphragmatic excursion and that measurements are influenced by body position.

The findings of the present study have important clinical implications across multiple contexts, including respiratory physiotherapy, sports medicine, functional research, and critical care. Identifying trunk inclinations that maximize diaphragmatic excursion in healthy individuals, particularly the 45° position as a global reference, provides a rational basis for standardizing assessment protocols, especially in populations with diaphragmatic dysfunction or weakness

Overall, these results expand our understanding of the diaphragm’s dynamic behavior in response to postural modifications and reinforce the relevance of body biomechanics in respiratory function. The variability observed in excursion according to trunk inclination angle demonstrates that the body does not respond uniformly to static positions, a fact that is particularly relevant in clinical settings where precision in functional assessment is required.

In this regard, the importance of establishing a standardized position for ultrasonographic measurement of diaphragmatic excursion is emphasized. Based on the present findings, a 45° trunk inclination emerges as the most appropriate standardized position for routine assessment, as it provides the most consistent global effect on diaphragmatic excursion. Higher inclinations, such as 70°, may be considered in specific contexts where sex-related differences are of interest. Relying solely on image quality criteria may lead to underestimation or overestimation of true diaphragmatic displacement, thereby compromising diagnostic accuracy or therapeutic follow-up.

This study not only provides relevant data for a healthy Latin American population but also highlights the importance of considering body positioning as a physiological and therapeutic tool to optimize respiratory function. Furthermore, it reinforces the value of M-mode diaphragmatic ultrasonography as a functional assessment method that is non-invasive, accessible, and reproducible in clinical practice.

Although the sample size was limited to 36 participants, the findings establish a preliminary foundation for future studies that may include populations with specific clinical conditions and explore additional variables such as age, sex, body composition, muscle fatigue, or the presence of chronic respiratory diseases. This approach would allow deeper exploration of the interaction between posture and diaphragmatic dynamics in more complex clinical scenarios.

In summary, the results presented here are not intended to establish definitive reference values but rather to provide a valuable starting point for the systematic study of diaphragmatic behavior in response to postural changes, encouraging the integration of the “body position” variable into functional respiratory assessment from a more applied, physiological, and contextualized perspective.

The strengths of this investigation lie primarily in its controlled methodological design and the use of M-mode diaphragmatic ultrasonography, currently considered the reference technique for assessing diaphragmatic excursion due to its high sensitivity and specificity [4]. Additionally, the study included a homogeneous sample of healthy adults, allowing control of clinical variables that could alter diaphragmatic function. Another important strength was the systematic evaluation across multiple body positions (0°, 30°, 45°, 70°, and 90°), providing a broad comparative perspective on postural influence. Finally, the pioneering nature of this study in a healthy Latin American population represents a valuable contribution, offering preliminary data that may serve as a reference for future investigations and cross-cultural comparisons.

Among the study limitations, the relatively small sample size should be acknowledged. Although the standard deviations observed for diaphragmatic excursion were higher than those assumed during the initial sample size estimation, the identification of statistically significant effects using a linear mixed-effects model suggests that the sample size was sufficient to detect relevant postural differences. Nevertheless, this increased variability should be interpreted within the exploratory nature of the study, which was not intended to establish definitive reference values. In addition, variables such as body composition, abdominal perimeter, and stratified body mass index were not evaluated, factors that may also influence diaphragmatic function [5]. Finally, the use of a single ultrasound window (right subcostal) may have limited bilateral hemidiaphragm assessment and restricted side-to-side comparisons.

The fixed and progressive order in which body positions were adopted (from 0° to 90°) represents an additional limitation of this study, as it may have introduced potential order effects such as adaptation, learning, or gradual changes in the respiratory pattern over time. Although randomization or counterbalancing of positions was not implemented, methodological strategies were applied to mitigate possible carryover effects. Specifically, a two-minute waiting period was allowed after each positional change before initiating measurements, to facilitate physiological accommodation and stabilization of ventilation and intra-abdominal pressure. During this interval, respiratory frequency stability was reconfirmed prior to image acquisition. This approach is supported by previous studies in individuals without underlying pulmonary disease who underwent abdominal surgery, in whom respiratory interventions or postural changes assessed using electrical impedance tomography demonstrated that ventilation distribution and intrapulmonary volume return to baseline conditions within approximately two minutes Nevertheless [21], these measures cannot fully eliminate potential order effects, and therefore the lack of randomization should be considered when interpreting the results. Future studies should incorporate randomized or counterbalanced position sequences to further minimize order-related bias and strengthen internal validity.

Building upon these methodological considerations, further research should also expand the scope of analysis to include additional individual characteristics. Future studies are recommended to incorporate variables such as body mass index, intra-abdominal fat percentage, and lean mass in order to establish more specific reference values and to better understand interindividual variability in diaphragmatic function across different clinical and functional contexts. Moreover, extending this research to critically ill populations would allow validation of the present findings under more complex physiological conditions.

## 5. Conclusions

The results of this study demonstrate that changes in body position significantly influence diaphragmatic excursion in healthy individuals. A trunk inclination of 45° showed the greatest overall effect on diaphragmatic excursion, while a 70° inclination exhibited a sex-dependent effect, with greater excursion observed in men. These findings support the importance of considering body posture as a key determinant in the functional assessment of the diaphragm using ultrasonography.

## Figures and Tables

**Table 1:** Sociodemographic and anthropometric characteristics.

Variable	Female (*n* = 18)	Male (*n* = 18)	Total (*n* = 36)	*p*-Value *
Age $\overset{-}{x}$ ± SD	41.4 ± 12.3	41.3 ± 14.8	41.2 ± 14.2	0.982
Socioeconomic status				
Low (1–2)	7 (38.9%)	1 (5.6%)	8 (22.2%)	
Middle (3–4)	8 (44.4%)	16 (88.8%)	24 (66.7%)	
High (5–6)	3 (16.7%)	1 (5.6%)	4 (11.1%)	0.013
Physical activity level				
Low	17 (94.4%)	14 (77.8%)	31 (86.1%)	
Moderate	1 (5.6%)	4 (22.2%)	5 (13.9%)	0.338
BMI $\overset{-}{x}$ ± SD	26.4 ± 4.1	26.8 ± 3.8	26.6 ± 3.9	0.763
Chest circumference $\overset{-}{x}$ ± SD	79.5 ± 8.6	85.8 ± 10.3	82.6 ± 9.8	0.045

Abbreviations: $\overset{-}{x}$, mean; SD, standard deviation; BMI, body mass index. * Chi-square test for differences in proportions and Student’s *t*-test for differences in means.

**Table 2:** Mean diaphragmatic excursion, SD, and 95% CI.

Body Position	Mean (cm)	SD (cm)	95% CI (cm)
0°	1.3647	0.37134	1.2391–1.4904
30°	1.4825	0.39701	1.3482–1.6168
45°	1.5736	0.45410	1.4200–1.7273
70°	1.7058	0.49383	1.5387–1.8729
90°	1.3906	0.36262	1.2679–1.5133

**Table 3:** Diaphragmatic excursion comparison between women and men.

Body Position	Women: Mean (cm) ± SD (cm) [95% CI]	Men: Mean (cm) ± SD (cm) [95% CI]
0°	1.37 ± 0.44 [1.15–1.59]	1.36 ± 0.30 [1.21–1.51]
30°	1.55 ± 0.49 [1.30–1.79]	1.42 ± 0.28 [1.28–1.56]
45°	1.59 ± 0.47 [1.35–1.82]	1.56 ± 0.45 [1.34–1.78]
70°	1.54 ± 0.49 [1.30–1.78]	1.87 ± 0.45 [1.65–2.10]
90°	1.32 ± 0.35 [1.15–1.50]	1.46 ± 0.37 [1.27–1.64]

## Data Availability

The original contributions presented in this study are included in the article. Further inquiries can be directed to the corresponding author.
